# 1020. Case Study. A Team Approach: Managing Cultured Epidermal Autograft While Undergoing Extracorporeal Life Support

**DOI:** 10.1093/jbcr/irag033.381

**Published:** 2026-04-06

**Authors:** Kara M Kastner

**Affiliations:** Medical City Plano, Dallas, TX

## Abstract

**Patient Presentation (age range, injury details, relevant history):**

Cultured epidermal autograft (CEA) is considered a life-saving measure for those with large total body surface area burns while extracorporeal life support (ECMO) is considered a life-saving measure for those with refractory cardiac or respiratory failure. However, the application of CEA on patients undergoing ECMO is not well studied. A single case study review was performed on a patient who sustained a 47% total body surface area flame burn with a grade three inhalation injury that successfully underwent CEA while on ECMO with the support of a team approach.

**Clinical Challenges:**

The patient was located on the cardiothoracic intensive care unit (CVICU) while receiving ECMO support. Preceding the surgical application of the CEA, a collaborative approach was taken amongst the burn trauma intensive care unit (BTICU) and CVICU nursing leadership to establish the pre and post-operative plan for the management of the patient.

**Management Approach:**

The Burn Educator (BE) attended unit huddles preceding the CEA application to provide the CVICU nurses education on CEA. Post-operatively, hand-outs were placed on the wall above the patients head and outside the patient’s door depicting where the grafts were placed and where the care team could touch the patient to eliminate graft loss/shearing. On the day of CEA application, the CVICU nurses traveled to and cared for the patient in the burn operating room (OR) amongst the other Burn OR team members. The CEA was applied to the patient’s anterior and posterior bilateral upper extremities with a 4:1 autograft underlay. CEA was also applied to the patient’s left calf donor site. Post-operatively, during dayshift, the patient was being cared for in a 3:1 assignment. The assignment consisted of an ECMO support specialist, a CVICU nurse and a BTICU nurse that was floated to the CVICU unit to ensure all the patients burn care needs were met. The Burn Nurse Supervisor (BNS), BE, Burn Wound Care Tech, and the assigned nurses worked together each day to remove the dressing and dry the patient out on arm supports. On POD 7, the BE, BNS, and Burn Advanced Practice Providers removed the dry-veil at bedside and the patient continued to dry-out until POD 12.

**Outcomes:**

On POD 12, it was determined by the burn surgeon that the patient’s right arm had 80% graft take and the left arm had 90% graft take. Ultimately, two weeks later, the family decided to withdraw care on the patient and they died.

**Lessons Learned:**

In this single case study review, it was concluded that with a team approach in mind, proper education,

tools, collaboration, and staffing with multiple nursing specialties, CEA graft take can be successful on a patient undergoing ECMO.

**Applicability to Practice:**

More studies are needed to determine the success rate of CEA while undergoing ECMO in order to best determine how to manage burn care when the patient is housed on a non-burn specialty unit.

